# Age-related modulation of plasmatic beta-Galactosidase activity in healthy subjects and in patients affected by T2DM

**DOI:** 10.18632/oncotarget.21848

**Published:** 2017-10-16

**Authors:** Liana Spazzafumo, Emanuela Mensà, Giulia Matacchione, Tiziana Galeazzi, Lucia Zampini, Rina Recchioni, Fiorella Marcheselli, Francesco Prattichizzo, Roberto Testa, Roberto Antonicelli, Paolo Garagnani, Massimo Boemi, Massimiliano Bonafè, Anna Rita Bonfigli, Antonio Domenico Procopio, Fabiola Olivieri

**Affiliations:** ^1^ Center of Biostatics, INRCA-IRCCS National Institute, Ancona, Italy; ^2^ Department of Clinical and Molecular Sciences, DISCLIMO, Università Politecnica delle Marche, Ancona, Italy; ^3^ Pediatric Division, Department of Clinical Sciences, Università Politecnica delle Marche, Ospedali Riuniti, Presidio Salesi, Ancona, Italy; ^4^ Center of Clinical Pathology and Innovative Therapy, INRCA-IRCCS National Institute, Ancona, Italy; ^5^ Department of Cardiovascular and Metabolic Diseases, IRCCS Multimedica, Sesto San Giovanni, Italy; ^6^ Clinical Laboratory and Molecular Diagnostics, INRCA-IRCCS National Institute, Ancona, Italy; ^7^ UTIC-Cardiology INRCA-IRCCS, National Institute, Ancona, Italy; ^8^ Department of Experimental, Diagnostic and Specialty Medicine, Alma Mater Studiorum, University of Bologna, Bologna, Italy; ^9^ Clinical Chemistry, Department of Laboratory Medicine, Karolinska Institutet at Huddinge University Hospital, Stockholm, Sweden; ^10^ Diabetology Unit, INRCA-IRCCS, National Institute, Ancona, Italy; ^11^ Scientific Direction, INRCA-IRCCS, National Institute, Ancona, Italy

**Keywords:** cellular senescence, beta galactosidase activity, type 2 diabetes, aging, inflammaging, Gerotarget

## Abstract

β-Galactosidase (β-Gal) activity has been the most extensively utilized biomarker for the detection of cellular senescence. It can be measured also in plasma, and few recent evidence showed an altered plasmatic β-Gal activity in patients affected by some age-related diseases (ARDs). Since T2DM is one of the most common ARDs, we aimed to investigate if plasmatic β-Gal activity is modulated in T2DM patients and if “age” could affect such modulation. To gain mechanistic insights we paralleled this investigation with the evaluation of β-Gal activity in young and senescent endothelial cells (HUVECs) cultured in normo- and hyper-glycaemic environment.

A significant age-related increase of plasmatic β-Gal activity was observed in healthy subjects (n. 230; 55-87 years), whereas the enzymatic activity was significantly reduced in T2DM patients (n. 230; 55-96 years) compared to healthy subjects.

β-Gal activity detectable both in cells and in the culture medium was significantly increased in senescent cells compared to the younger ones, both under normo- and hyper-glycaemic condition. However, the hyper-glycaemic condition was not associated with an increased β-Gal activity in milieu compared to normo-glycaemic condition.

Overall our data reinforce the notion that plasmatic β-Gal activity could be a systemic biomarker of aging, whereas T2DM patients are characterized by a different age-releated trend.

## INTRODUCTION

The human lysosomal enzyme β-D-galactosidase (β-Gal) is an exoglycosidase that catalyzes the hydrolysis of terminal β-linked galactose residues in glycoproteins, glycolipids and proteoglycans [1]. A deficiency of lysosomal acid β-Gal activity detectable in blood cells, *i.e.* leukocytes, and biological fluids, *i.e.* plasma, is currently a diagnostic biomarker for some rare inherited lysosomal storage diseases, such as GM1-gangliosidosis and Morquio B disease [2]. Increasing evidence support the view that the integrity of the autophagosomal-lysosomal network is critical in the progression of aging [3]. This hypothesis is reinforced by the evidence that lysosomal dysfunctions are associated with the onset and progression of many age-related diseases (ARDs), including Parkinson’s and Alzheimer’s disease (AD) [4, 5] and type 2 diabetes (T2DM) [6, 7]. Altered transcriptional and translational levels of several lysosomal glycohydrolases and proteases, including β-Gal, was observed in skin fibroblasts, leukocytes and post-synaptic vesicles of patients affected by AD [7-10]. Increased β-Gal activity in leukocytes was observed also in patients affected by Down’s syndrome (DS), patients with an increased risk of develop many age-related chronic diseases [9, 11]. Although in the past little attention was paid to glycohydrolases present in cellular compartments different from lysosomes, growing evidence suggested the presence of active lysosomal enzymes in extra-lysosomal compartments, such as the plasma membrane [12] and the extracellular environment [13]. Autophagolysosomes and their content instead of being fully processed by degradation can be extruded from cells through unconventional secretion mechanisms, including the so called “secretory autophagy” [14, 15]. The link between β-Gal and aging is reinforced by the identification of a β-Gal activity in senescent cells, which has been named senescence-associated (SA)-β-Gal [16]. The increased SA-β-Gal activity observed in senescent cells seems due, at least partly, to the increased expression of the lysosomal β-Gal protein [17]. SA-β-Gal activity has been the most extensively utilized biomarker for the detection of cellular senescence both *in vitro* and *ex vivo* [18-22]. However, the remarkable asynchrony and heterogeneity of cellular senescence remain a challenge for investigating the relationship between the number of senescent cells, the rate of aging, and the risk of ARDs. The research of circulating biomarkers to measure the “systemic senescence status” is a cutting-edge problem. Plasmatic β-Gal activity can be measured, and some studies showed a differential plasmatic β-Gal activity in patients affected by AD and T2DM [7], as well as in many types of cancers [23, 24]. It was therefore hypothesized that plasmatic β-Gal activity might be a manifestation of “systemic senescence status” in the course of ARDs [25].

A significant shift in serum N-glycan profile was observed during ageing. The logarithm of the ratio between the agalactosylated glycan (NGA2F) and the galactosylated glycan (NA2F) showed a strong correlation with age and therefore it was named “GlycoAgeTest” [26]. Almost all secreted proteins are post-translationally modified with the covalent attachment of N-glycans, and the β-Gal and others exoglycosidases are involved in the remodelling of N-glycan structures, including NGA2F and NA2F [26, 27]. We previously observed specific N-glycan profiles in T2DM patients [28].

Since T2DM is one of the most common age-related diseases and no data are currently available on plasmatic modulation of β-Gal activity during aging and no studies have already evaluated the association between GlycoAgeTest and plasmatic β-Gal activity, we aimed to investigate these associations. To gain mechanistic insights we aimed to parallel the investigation on plasma samples with the evaluation of β-Gal activity both inside and outside young and senescent endothelial cells (HUVECs) cultured in normo-glycaemic and hyper-glycaemic environment, the latter ones mimicking the main diabetes pathological feature.

## RESULTS

### Age-related trend of plasmatic β-Gal activity in healthy subjects and in T2DM patients

The anthropometric and biochemical parameters of 230 healthy subjects, defined as control subjects (CTR) and 230 patients affected by T2DM (defined as T2DM) were reported in Table 1A and 1B, respectively. Since we aimed to investigate the age-related correlations of the selected parameters we grouped the subjects in three different age-classes such as: first group: ≥55 and <65, second group: ≥65 and <75 years and third group: ≥75 years. These cut-offs are appropriate for considering a subject to be elderly, as reported in previous studies [29, 30].

**Table 1:** Anthropometric and biochemical parameters. (A) Parameters of healthy subjects (n. 230).

Parameters	Healthy subjects (n. 230)			
	≥55 and <65 years (n. 70)	≥65 and <75 years (n. 115)	≥75 years (n. 45)	P
BMI, kg/m^2^	27.71± 4.23	27.26 ± 4.33	27.26 ± 4.33	0.959
HDL, mmol/L	54.15± 13.36	54.91 ± 16.78	53.28 ± 14.09	**0.021**
LDL, mmol/L	131.28 ± 36.09	128.04± 31.77	128.08 ± 28.88	0.868
TC, mmol/L	220.35±37.87	223.25±38.10	214.99±39.02	0.605
FG, mg/dl	98.91±9.6	93.60±10.51#	94.80 ±10.91	0.071
HbA1c	5.78±0.38	5.69±0.35	5.74±0.45	0.677
WC,	6.37±1.52	6.04±1.44	5.88±1.41	0.321
CRP	2.43±3.21	5.54±7.88#	5.59±10.77#	**0.001**
Creatinine	0.86±0.19	0.83±0.20	0.86±0.24	0.775
GlycoAgeTest	-0.28±0.13	-0.22±0.13#	-0.18±0.13#*	**<0.001**
Plasmatic β-Gal activity (nM/ml/hr)	5.17±2.60	5.49 ± 3.68	6.95 ± 3.08*	**0.016**

^#^ p<0.05, comparison with ≥55 and <65 years; * p<0.05, comparison with ≥65 and <75 years. P from ANOVA. Significant values were reported in bold. TC = total cholesterol; FG=fasting glucose; HbA1c= Glycated haemoglobin; WC=white cells; CRP=C Reactive Protein; GlycoAge Test=Log (CC1/CC6).

**Table 1:** Anthropometric and biochemical parameters. (B) Parameters of T2DM patients (n.230)

Parameters	T2DM (n. 230)			
	≥55 and <65 years (n. 112)	≥65 and <75 years (n. 88)	≥75 years (n. 30)	P
BMI, kg/m^2^	29.88±3.78	28.97±4.06	26.77±2.84	0.249
LDL, mmol/L	122.45±32.76	121.34±31.22	111.34±30.21	0.581
HDL, mmol/L	52.18±16.51	54.08±15.31	56.66±19.22	0.516
TC, mmol/L	211.66±39.66	209.78±37.92	212.76±36.88	0.923
FG, mg/dl	175.77±53.42	164.65±47.22	168.88±53.76	0.631
HbA1c	7.65±1.34	7.34±1.08	7.48±1.23	0.355
WC	6.82±1.78	6.54±1.37	6.21±1.17	0.490
CRP	4.66±6.33	3.45±4.22	3.88±2.75	0.710
Creatinine	0.86±0.27	0.93±0.36	1.21±0.55*	**0.007**
GlycoAgeTest	-0.28±0.15	-0.22±0.15#	-0.23±0.15#	0.146
Plasmatic β-Gal activity (nM/ml/hr)	4.79±3.88	3.88±3.38	4.46±3.07	0.157

^#^ p<0.05, comparison with ≥55 and <65 years; * p<0.05, comparison with ≥65 and <75 years. P from ANOVA. Significant values were reported in bold. TC = total cholesterol; FG=fasting glucose; HbA1c= Glycated haemoglobin; WC=white cells; CRP=C Reactive Protein; GlycoAge Test=Log (CC1/CC6).

The mean age of each group of subjects was about 60, 70 and 80 respectively.

Significant increased values of plasmatic β-Gal activity were observed in elderly CTR compared with younger ones (Table 1A).

Significant increase of C-reactive protein (CRP) mean value was observed in groups of 70 and 80 years compared to that of 60 years (Table 1A), whereas GlycoAge Test values decreased significantly among the three groups (Table 1A).

Significant age-related trends were confirmed by correlation analysis for plasmatic β-Gal activity (Pearson’s correlation 0.24, p<0.01), GlycoAge Test (Pearson’s correlation 0.25, p<0.01) and CRP (Pearson’s correlation 0.24, p<0.01), (Table 2).

**Table 2:** Correlation between plasmatic parameters and age of healthy subjects. Healthy subjects, n=230

	healthy subjects (n. 230)	
	Pearson’s correlation	p
FG, mg/dl	-0.12	0.07
CRP	**0.24**	**<0.01**
GlycoAgeTest	**0.25**	**<0.01**
β-Gal	**0.24**	**<0.01**

In patients affected by T2DM no significant age-related trend were observed for any analysed variables including β-Gal activity (Pearson’s correlation: -0.12, p=0.07).

However, GlycoAge Test value was significantly reduced in groups of older patients (Table 1B) and a significant increase of creatinine mean value was observed in the group of the oldest T2DM patients (Table 1B).

**Figure 1:**
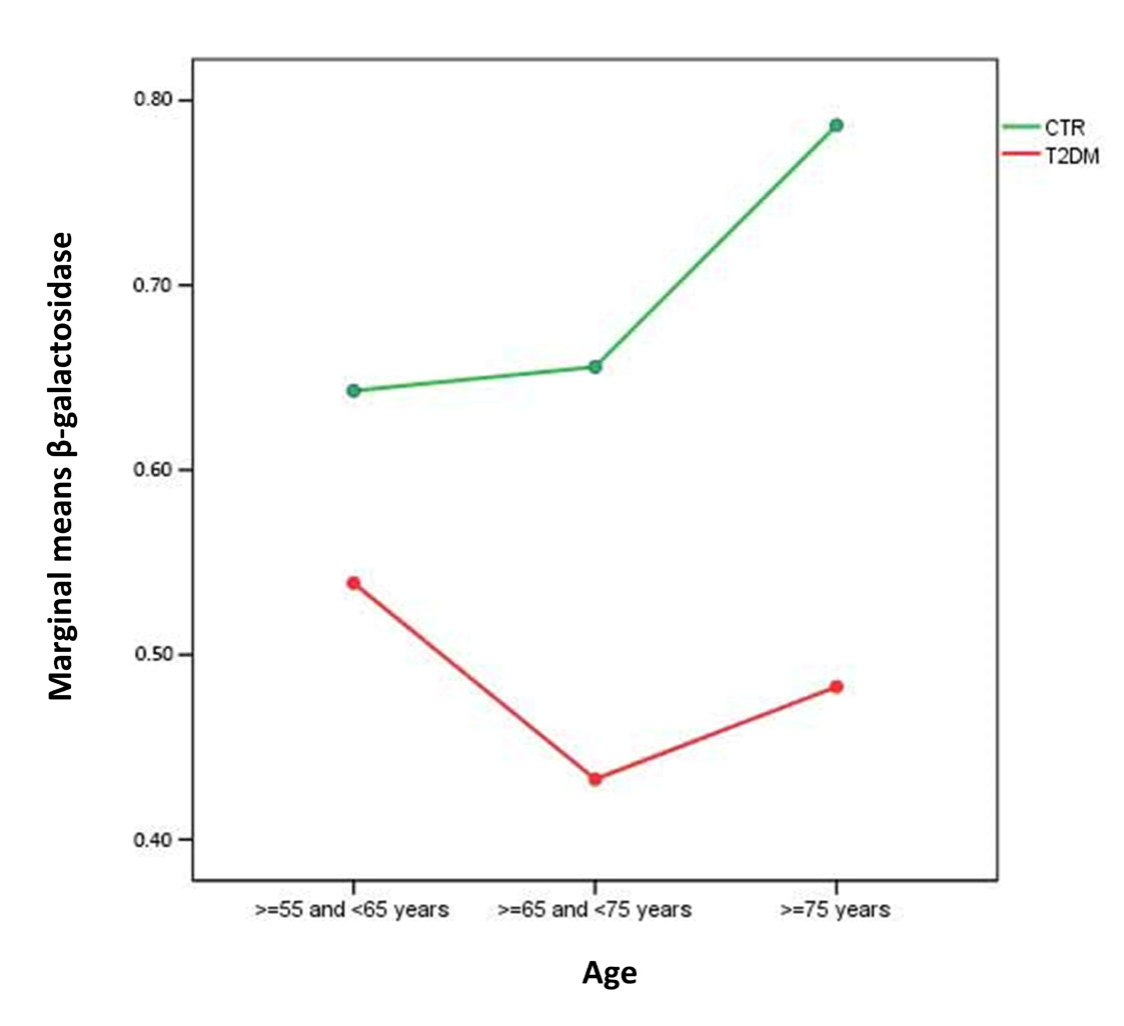
Marginal means of β−Gal activity in healthy subjects and T2DM patients groups Healthy subjects, green line, n=230; T2DM patients, red line, n=230. First group: ≥55 and <65, second group : ≥65 and <75 years and third group : ≥75 years.

When age-related trend of β-Gal activity was compared between healthy subjects and T2DM patients a significant difference was unravelled (ANOVA, F =4.916, p=0.027). Marginal means of β−Gal activity in healthy subjects and T2DM patients groups were shown in green and red respectively in Figure 1.

We then analysed the correlation between β-Gal and the others variables significantly related to age. No significant correlations were observed between plasmatic β-Gal activity and GlycoAge Test in healthy subjects (data not shown). Therefore, to unravel the relative contribution of GlycoAge Test and β-Gal as age-related variables of healthy status, we performed a sequential linear regression analysis, including age as dependent variables and GlycoAge Test, β-Gal, CRP, HDL, and fasting glucose (FG) as independent variables. In the first step of regression analysis a significant correlation between β-Gal and age was confirmed (beta coefficient= 0.23, p<0.001l), as well as between GlycoAge Test and age (beta coefficient= 0.24, p<0.001). In the second step, all the parameters significantly related with age, including FG, were included as independent variables, confirming the significant correlation between β-Gal and age (beta coefficient= 0.21, p=0.001 for β-Gal and beta coefficient= 0.20, p<0.002 for GlycoAge Test).

**Figure 2:**
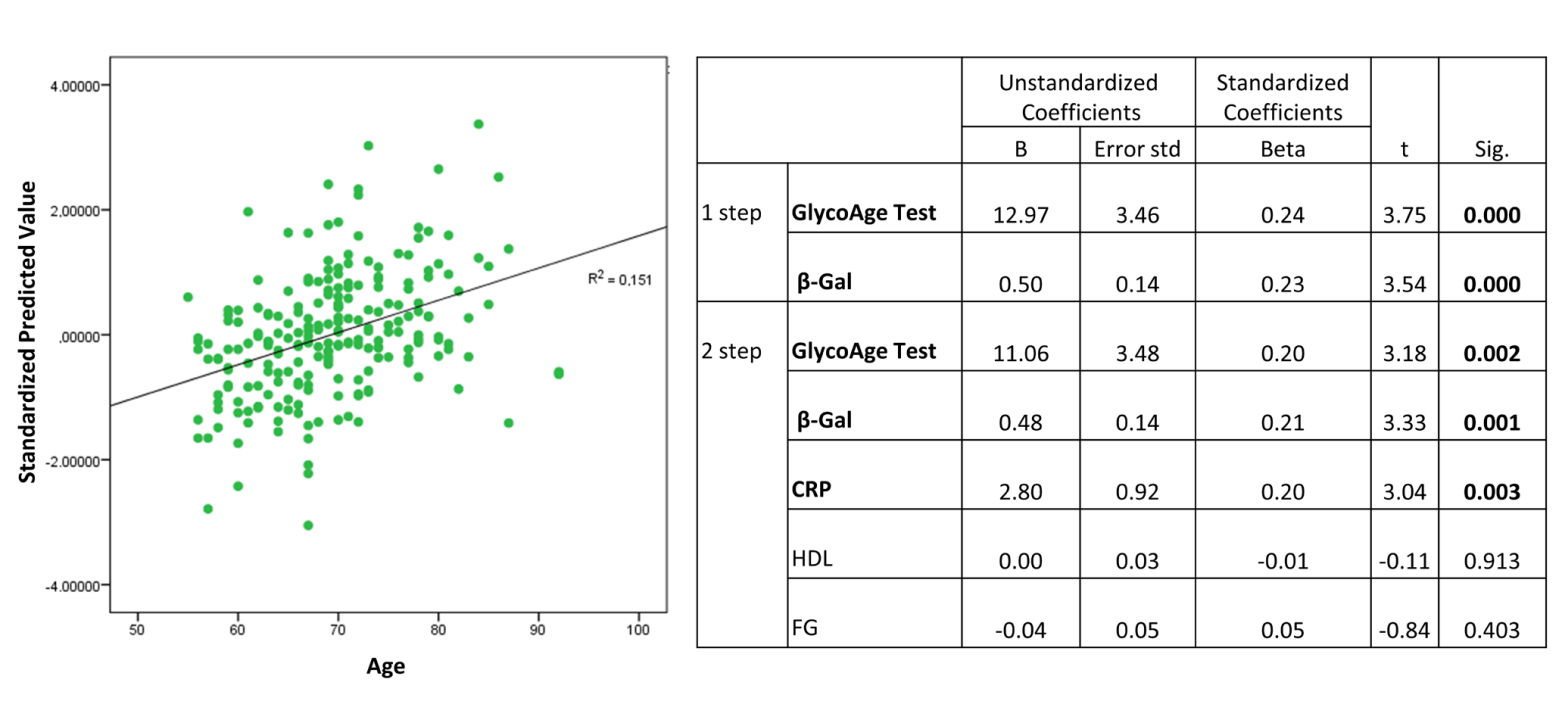
Standardized predicted values of β-Gal activity in plasma from healthy subjects Plasma from healthy subjects, n=230.

The sequential multiple regression revealed that, at step one, GlycoAge Test and β-Gal contribute significantly to the regression model (F test = 13.85, p< 0.001) and account for about 11% of the variation in Age. The addition of the others parameters, such as HDL, CRP and FG to the regression model, explains an additional 4.1% of the variation in Age (Figure 2, R squared =0.15).

Standardized predicted β−Gal activity values for healthy subjects were shown in Figure 2.

### Comparison of plasmatic β-Gal activity between T2DM patients and age-matched CTR

**Figure 3:**
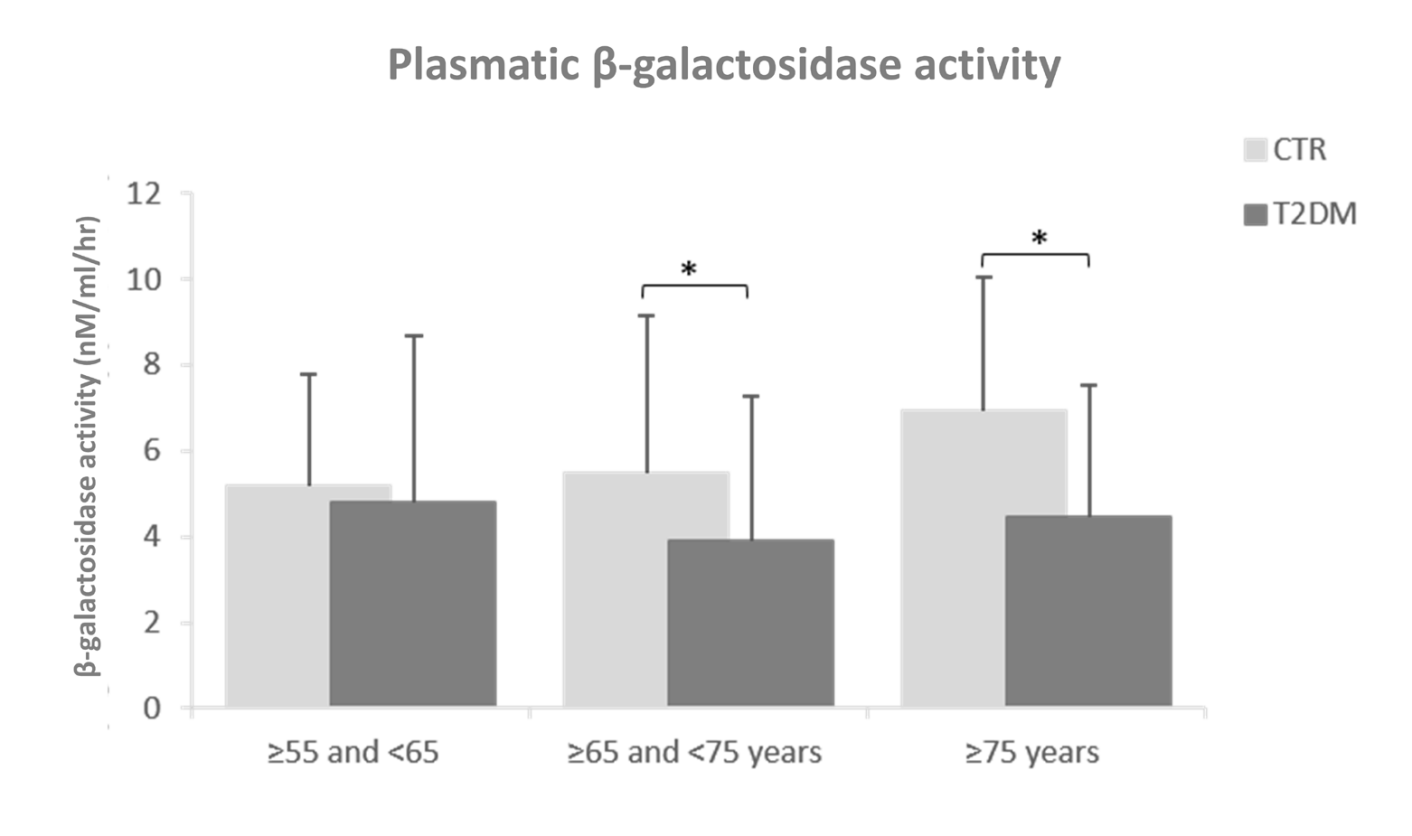
Plasmatic β-Gal activity in T2DM patients compared to age-matched healthy subjects Healthy subjects, n=230; T2DM patients, n=230. First group: ≥55 and <65, second group : ≥65 and <75 years and third group : ≥75 years. Plasmatic β-Gal activity was reported as nM/ml/hr.

Plasmatic β-Gal activity was significantly decreased in T2DM patients aged more than 65 years compared to age-matched healthy subjects (Figure 3). Linear regression analysis did not identified parameters with significant age-association in T2DM patients (data not shown).

Plasmatic β-Gal activity was not significantly different among the three age-groups of T2DM patients according to the presence of at least one complication (data not shown). When the three age-groups of T2DM patients were grouped according to HbA1c cut-off level (7%), plasmatic β-Gal activity was not significantly different (data not shown). Finally, when T2DM patients were grouped according to metformin or sulfonylurea treatments, plasmatic β-Gal activity was not significantly different among the three age-groups (data not shown).

### β-Gal activity in young and senescent endothelial cells

**Figure 4:**
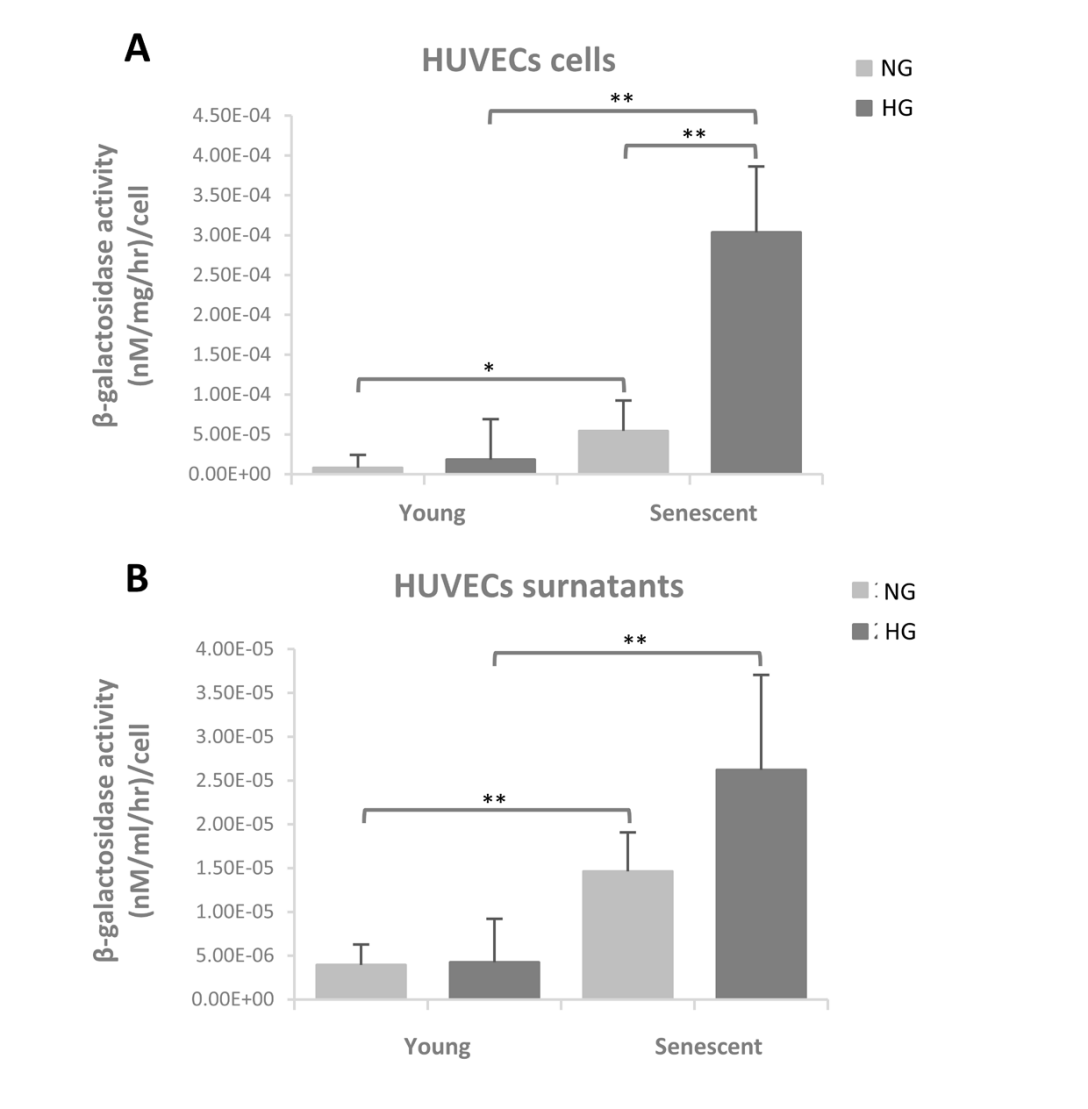
β-Gal activity evaluated in young and senescent HUVECs cultured both in normoglycaemic and hyperglycaemic milieu (**A**) β-Galactosidase activity in cells reported as (nM/mg/hr)/cell (**B**) β-Galactosidase activity in supernatants reported as (nM/ml/hr)/cell. Data are presented as mean and standard deviation (SD) of three independent experiments. Hyperglycaemic milieu: glucose 25 mM.

β-Gal activity was evaluated in young and senescent HUVECs cultured both in normo- and hyper-glycaemic milieu. β-Gal activity was evaluated in cells and in supernatants, in order to obtain data on the enzyme release. A significant 4.8-fold increased activity was observed in senescent cells compared to the younger ones in normo-glycaemic condition whereas in hyper-glycaemic condition β-Gal activity increased 11-fold in senescent cells *vs.* younger cells (Figure 4A). In senescent HUVEC cultured in hyper-glycaemic medium β-Gal activity was also significantly increased in comparison with senescent cells cultured in normo-glycaemic condition (Figure 4A).

β-Gal activity detectable in the culture medium was significantly increased in senescent cells compared to the younger ones, both under normo- and hyper-glycaemic milieu (Figure 4B). However, the hyper-glycaemic condition is not associated with an increased β-Gal activity in milieu compared to normo-glycaemic condition (Figure 4B).

## DISCUSSION

This is the first report demonstrating a significant age-related increase of plasmatic β-Gal activity in healthy subjects. β-Gal activity is the most widely used biomarker for the identification of senescent cells and our results can be considered a “proof of principle” that plasmatic β-Gal activity could be a non-invasive surrogate biomarker able to track senescent cells burden at systemic level. The relevance of our result is related to the evidence that the delaying of senescent cell accumulation or the reduction of senescent cell burden is associated with delay, prevention, or alleviation of multiple senescence-associated conditions and ARDs [31]. Faithful meters are needed to estimate the effects of intervention based on recently discovered molecules able to modulate the senescence process.

When we measured β-Gal activity in plasma of T2DM patients of different age, we observed that the enzymatic activity was significantly reduced in T2DM patients compared to CTR, confirming a previous result [7]. Considering T2DM patients as subjects with an accelerated systemic senescence, this is a counterintuitive result [32-34]. Different phenomena could explain the reduced plasmatic β-Gal activity in T2DM patients: 1- diabetic state could worsen lysosomal dysfunction [35]; 2- chronic hyperglycaemia/diabetic status could induce a number of imbalances in enzymes involved in hexoses metabolism, including galactosidases [36]; 3- an increased release of proteins derived from lysosomes and vascular epithelium into the urine may result from hyperglycemia-associated inflammation in the kidney vasculature, as recently suggested in patients affected by T1DM [37]; 4- hyperglycaemia could modulate the secretory abilities of cells. To verify this latter hypothesis, we measured β-Gal activity in young and senescent HUVECs and in their supernatants. We observed a significant increased β-Gal activity in senescent cells compared to younger ones, confirming our previous results on SA- β-Gal in HUVECs [38].

In normo-glycaemic condition β−Gal activity measured in *in vitro* vs. *ex vivo* models showed concordant results, suggesting that there is a significant increasing age- and senescence-related trend. In hyper-glycaemic condition β−Gal activity measured in *in vitro* vs. *ex vivo* models showed discordant results, since the decreasing trend observed *in ex vivo* was not confirmed in *in vitro* experiments on endothelial cells. These apparently conflicting results could be explained considering that tissues different from endothelium could contribute to circulating β-Gal activity and/or that pharmacological therapies could affect plasmatic β-Gal activity. When T2DM patients were grouped according to metformin or sulfonylurea treatments, plasmatic β-Gal activity was not significantly different among the three age-groups, suggesting that it was not affected by hypoglycemic agents. However, we cannot exclude that pharmacological therapies different from metformin and sulfonylurea (*i.e.* statins or beta-blockers) could affect plasmatic β-Gal activity. Further studies including more detailed information on pharmacological therapy could clarify this issue.

Moreover, since T2DM patients escape from the age-related trend of plasmatic β−Gal activity observed in healthy subjects, probably due to their consistent metabolic imbalances, further studies including patients affected by different ARDs are mandatory.

Notably, β-Gal and others exoglycosidase are involved in the remodelling of N-glycan structures [26, 27, 39, 40]. The GlycoAge Test, showed a strong correlation with chronological age [26, 41, 42]. Notably, the structures of the glycan linked to the plasmatic proteins (i.e. IgGs), modulate the pro- or anti-inflammatory activities of the proteins, because the glycoforms lacking terminal galactose are particularly proinflammatory, whereas the bigalactosylated glycans are anti-inflammatory [43]. Therefore, the progressive age-associated glycan changing is consistent with a shift toward a pro-inflammatory agalactosylated glycotype, that could contribute to fuel inflammaging, the chronic, systemic, low levels of inflammation involved in the development of the most common ARDs [44, 45].

A significant age-related increase of plasmatic β4-galactosyltransferase activity, involved in the transformation of NGA2F in NA2F glycan was recently reported [44], whereas the age-related trend of plasmatic β-Gal activity, involved in the transformation of NA2F in NGA2F, has never been investigated. We hypothesized a correlation between plasmatic β-Gal activity and agalactosylated plasmatic glycans levels.

However, we did not observe a significant correlation between the two parameters. Our results are in line with data obtained by Catera and colleagues [44], showing that glycosylation of plasmatic proteins is not correlated with the activity of the plasmatic glycosyltransferases.

Notably, β-Gal activity was detected at pH 4, but the activity of plasmatic β-Gal at physiological pH could be lower than that measured at pH 4. We cannot exclude that plasma β-Gal could be contained inside exosomes or others microvesicles and that the internal pH of these organelles could be different from that of plasma.

Overall our data reinforce the notion that plasmatic β-Gal activity detectable with a simple biochemical assay could be a systemic biomarker of aging, and should be analysed in association with the GlycoAgeTest. However, the molecular mechanisms involved in the shift toward a pro-inflammatory agalactosylated glycotype during aging, as well as the mechanisms involved in the release of lysosomal enzymes in plasma and their functions deserve further investigations.

## MATERIALS AND METHODS

### Subjects

This study involved subgroups of a cohort of 501 T2DM patients and 400 healthy subjects enrolled in the framework of an Italian national study to identify biological parameters associated with T2DM [46]. All the information collected and the inclusion criteria were as described in Testa [46].

Since T2DM is a well characterized age-related disease we selected only subjects with age ≥ 55 years. Among the 490 T2DM patients and 300 healthy subjects older than 55 years we selected for the present study 230 T2DM patients and 230 healthy subjects taking into account the availability of plasma samples. The age ranges were 55-87 years and 55-96 years for T2DM and healthy subjects respectively.

Of the 230 T2DM patients, 134 have at least one complication, such as retinopathy (n. 93), major adverse cardiac events (MACE) (n. 49), neuropathy (n. 41), nephropathy (n. 28), lower limb arteriopathy (n. 7) and chronic renal failure (n. 9). Each patient could have more than one complications.

### Cellular models

HUVECs were purchased from Clonetics Corporation (Lonza, Basel, Switzerland) and cultured in EGM-2 endothelial growth medium (Lonza). Briefly, fresh cells were seeded at a density of 2,500 cells/cm^2^ in T 75 flasks, the medium was changed every 48 h. Cultures reached confluence after 6-7 days, as assessed by light microscopic examination, and were passaged at weekly intervals. After trypsinization and before replating, harvested cells were counted using a haemocytometer. Replicative senescence was studied by culturing cells until the proliferative arrest, as described previously [47]. Viable cells were counted at each passage by trypan blue staining; population doublings (PDs) were determined as current PDs=last PDs+log2 (collected cell number / seeded cell number); cumulative population doubling (CPD) was calculated as the sum of all PD changes [38].

Cells were divided into young (CPD=20 ± 2) and senescent (CPD=46 ± 2). The characterization of senescence status was performed as described in Olivieri [38].

A hyperglycaemia-like environment was obtained by culturing young and senescent HUVECs in high glucose (25 mM) medium (HG) for 7 days.

### Laboratory assays

Glycaemia, glycated haemoglobin (HbA1c), total cholesterol, HDL, CRP and white blood cell (WC), were determined by standard automated procedures.

### Quantitative assay of β-Gal activity using endothelial cells and plasma samples

Endothelial cells or plasma β-Galactosidase activity was measured performing a fluorometric assay using 1mM 4-Methyl-umbelliferyl-β-D-galactopyranoside in citrate-phosphate buffer, pH 4.0 as substrate.

Endothelial cells pellet was re-suspended in a suitable amount of water and sonicated 3 times for 15” in ice-bath to obtain homogenous cells suspension. A quantitative estimation of total protein concentration was made using a colorimetric assay (Bio-Rad Protein Assay,) reading the optical density (OD) at 595 nm [48].

50 μL of plasma samples and a variable volume of cells suspension (μL) containing 20-30 μg of proteins, were incubated with 200 μL and 300 μL of the substrate solution, respectively. After 1 hour at 37°C, the reaction was stopped with 2.5 mL of glycine-carbonate buffer. The fluorescence of the liberated 4-Methylumbelliferone was measured on RF-1501 spectrofluorophotometer (Shimadzu) at excitation wavelength=360 nm and at emission wavelength= 446 nm.

Enzymatic activity was expressed in nM/mg/hr for endothelial cells and nM/ml/hr for plasma samples respectively [49].

### The GlycoAge Test

The N-linked glycans peaks 1 and 6 were previously analyzed [28], using DSA-FACE technology, as previously described [26]. Peak 1 is the NGA2F, whereas peak 6 is its bigalactosylated counterpart NA2F. The Log of the ratio of the relative abundance of peaks 1 and 6 was defined “GlycoAge Test” [26].

### Statistical analysis

Comparisons among groups were conducted with the analysis of variance (ANOVA) and covariance (ANCOVA) as appropriate.

Correlations between parameters were calculated using Pearson’s correlation coefficient or partial correlation coefficient (r) controlled for age.

Sequential regression was employed to determine if additional parameters could improve the prediction of age beyond that afforded by differences in GlycoAge Test and β-Gal in healthy subjects. A two steps multiple regression was conducted with Age as the dependent variable. GlycoAge Test and β-Gal were included in the analysis at step one, whereas the others parameters were entered at stage two of regression.

Data analysis was carried out with the SPSS/Win program version 22 (SPSS, Chicago, IL). Statistical significance was defined as a two-tailed p value < 0.05.
